# Deep learning of tautomer stability from crystallographic proton positions

**DOI:** 10.1039/d6sc03714c

**Published:** 2026-09-23

**Authors:** Xiaolin Pan, Chao Han, Fengyang Han, Yingkai Zhang

**Affiliations:** a Department of Chemistry, New York University New York 10003 USA Yingkai.zhang@nyu.edu; b Simons Center for Computational Physical Chemistry at New York University New York New York 10003 USA

## Abstract

Tautomerism plays a central role in molecular recognition, physicochemical properties, and chemical reactivity, yet rapid identification of stable tautomeric states remains a persistent challenge in molecular design and structure-based drug discovery. Quantum-mechanical approaches are often too computationally demanding for library-scale application, while rule-based and 3D-dependent machine-learning methods remain limited in either transferability or throughput. Here, we show that experimentally resolved hydrogen positions in the Cambridge Structural Database (CSD) provide a powerful large-scale source of supervision for learning tautomer stability. Using more than 1.1 million tautomeric states derived from proton-resolved crystal structures, we trained a 2D graph neural network that predicts stable tautomeric states directly from molecular topology, without conformer generation or quantum calculations. The model achieved a recall of 0.97 and a precision of 0.88 on a comprehensive test set spanning crystal structures, aqueous-solution benchmarks, and drug-like molecules, demonstrating encouraging performance across crystalline and aqueous environments. Applied to 5075 PDBbind ligands with multiple tautomeric states, the model identified 126 cases in which the assigned tautomeric state is likely incorrect; in each case, the reassigned stable tautomer exhibited improved hydrogen-bonding patterns together with fewer unsatisfied polar atoms. We further developed an open-source workflow, Tautomer-Predictor, capable of processing the 4.6-million-compound Enamine collection in about 3.2 h on a single GPU-enabled node. Together, these results establish crystallographic proton placement as a rich and underexploited source of chemical knowledge for learning tautomer stability, and provide a practical route to large-scale tautomer assignment for molecular discovery.

## Abstract 2

By mining crystallographic proton positions, we trained a graph neural network to predict stable tautomeric states directly from 2D molecular topology, enabling rapid tautomer assignment for high-throughput molecular discovery.

## Graphical abstract

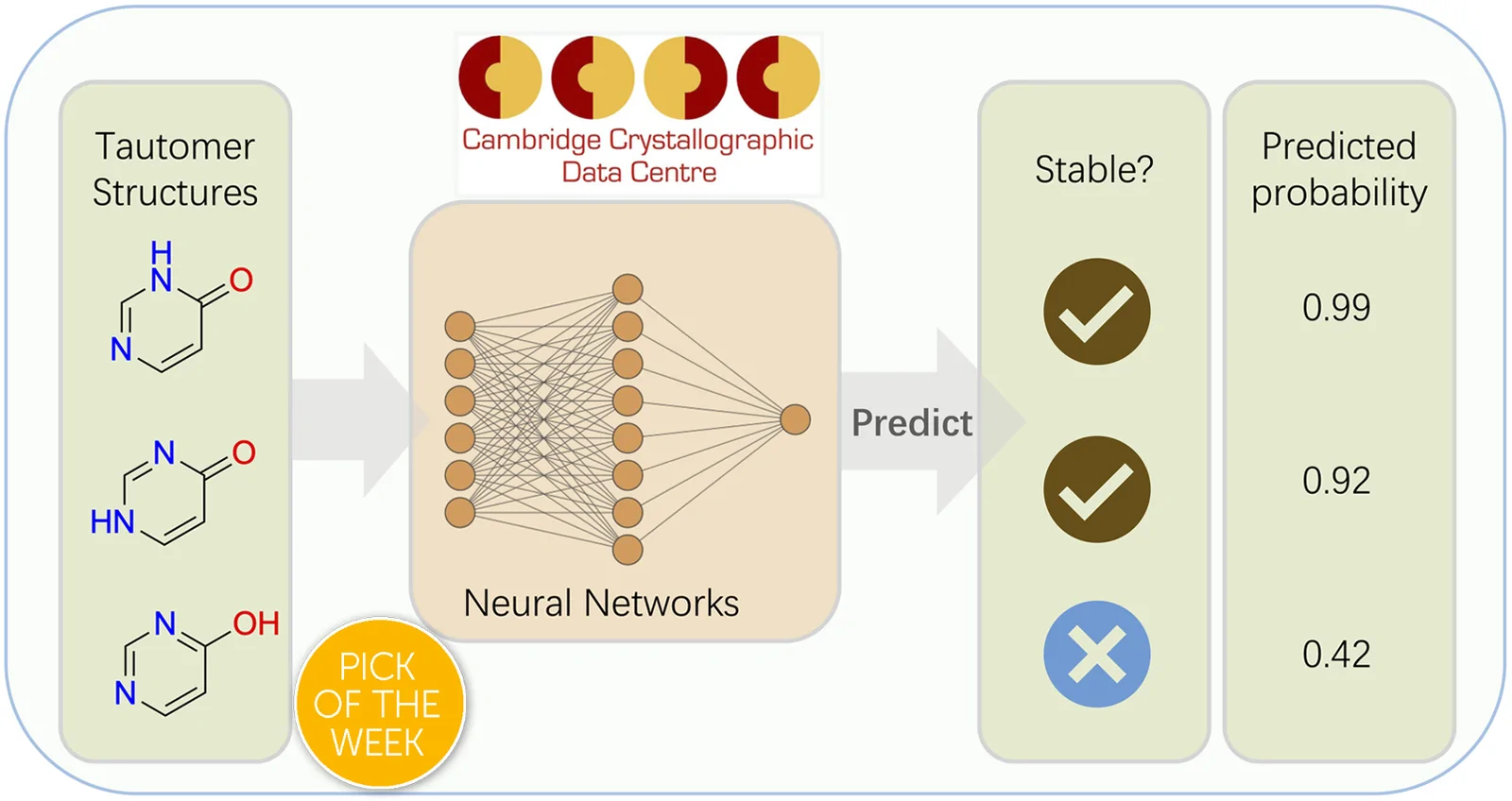

## Introduction

Tautomerism is a fundamental form of structural isomerism involving proton migration and bond rearrangement, and it plays a central role in molecular recognition, physicochemical properties, and chemical reactivity. In small molecules and biomolecular building blocks alike, the preferred tautomeric state can strongly influence p*K*_a_, solubility, lipophilicity, hydrogen-bonding patterns, and ultimately biological function.^1–5^ In medicinal chemistry, this is particularly important because tautomeric shifts can alter a ligand pharmacophore by interconverting hydrogen-bond donors and acceptors, thereby changing both target binding and structure-based modeling outcomes. As a result, incorrect tautomer assignment can compromise molecular docking, free-energy calculations, virtual screening, and other tasks in structure-based drug design.^6–12^ Despite its importance, rapid and reliable identification of stable tautomeric states remains challenging, especially across the large and chemically diverse molecular spaces relevant to modern drug discovery.

A variety of computational methods have been developed to address tautomer prediction, but each faces important trade-offs between accuracy, throughput, and generalizability. High-level quantum mechanics (QM) calculations can in principle provide physically meaningful tautomer energetics in solution, but their computational cost makes them impractical for screening large chemical libraries.^13–17^ Rule-based and empirical methods are much faster, yet they depend on predefined transformations or fragment-based heuristics derived from limited experimental or calculated data, which can reduce transferability to new chemotypes.^4,6,18–24^

Recently, advances in artificial intelligence have offered a promising alternative. Liu *et al.* developed Auto3D, which generates low-energy conformations and tautomeric states in the gas phase using the ANI-2xt deep potential.^25^ Ji *et al.* developed MolTaut, a hybrid method combining the ANI-2x potential with a solvent model learned from density functional theory (DFT) data to rank tautomeric states.^26^ A key limitation of this approach is the requirement for ANI-2x geometry optimization prior to energy calculation, which restricts its utility for high-throughput screening of large chemical libraries. In a different approach, Wieder *et al.*^27^ integrated molecular dynamics (MD) simulations with an alchemical free energy protocol to fine-tune the ANI-1ccx model, incorporating solvent effects for tautomer ratio prediction in aqueous solution. Although these approaches improve accuracy, their reliance on geometry optimization, molecular simulation, or free-energy calculations limits their utility for ultra-high-throughput screening. Our previous model, sPhysNet-Taut,^28^ improved the speed and accuracy of tautomer-ratio prediction in aqueous solution, but it still requires 3D conformations and depends on relatively limited experimental data for fine-tuning. Thus, there remains a clear need for a method that is simultaneously fast, experimentally grounded, and broadly transferable across chemical space.

In this work, we show that experimentally resolved hydrogen positions in the Cambridge Structural Database (CSD) provide a powerful large-scale source of supervision for learning tautomer stability. Because many CSD structures contain explicitly assigned hydrogen atoms, they offer direct experimental information about tautomeric states in the crystalline environment. By mining these proton-resolved structures, we constructed a dataset of more than 1.1 million tautomeric states and trained a 2D graph neural network that predicts stable tautomeric states directly from molecular topology, without conformer generation or quantum-mechanical calculations. We then evaluated the model across crystal-structure, aqueous-solution, and drug-like benchmarks to test both predictive performance and transferability beyond the crystalline environment. Finally, we developed an open-source workflow, Tautomer-Predictor, and applied it to the PDBbind v2020 dataset and several large compound collections to assess its practical value for structure-based modeling and library-scale molecular screening.

## Materials and methods

### Dataset

To train and fully evaluate our model, we used the CSD database,^29,30^ Tautobase,^31^ the Tautomer Database,^32^ DrugBank,^33^ and LigandExpo^34^ to construct a training set, a validation set, and five test sets: (i) a multi-tautomer set from crystal structures; (ii) a 100-tautomer set from aqueous solution; (iii) a tautomer-mix set from water or water-mixture solvents; (iv) an overlap set from the intersection of CSD, DrugBank, and LigandExpo; and (v) a drug-like set, which is larger than the overlap set and is derived from the intersection of CSD with DrugBank or LigandExpo. The sources and sizes of these datasets are described in Table 1. The overall dataset construction workflow is illustrated in Fig. 1A. For each molecule, all possible tautomers were enumerated using RDKit, designating the CSD tautomer as the stable form and the remaining enumerated forms as unstable (as shown in Fig. 1B). This labeling strategy inevitably introduces some noise into the dataset. While certain molecules can exist as multiple stable tautomers, a given CSD entry captures only one specific tautomeric state. Because our workflow strictly defines the CSD-observed state as stable and all other RDKit-generated forms as unstable, some viable tautomers may be mislabeled as unstable. Nevertheless, the large scale of the CSD-derived dataset and the prevalence of experimentally observed low-energy tautomeric states should allow the model to learn robust chemical patterns associated with tautomer stability.

**Table 1:** Datasets constructed in this study, including the training set, validation set, multi-tautomer set, 100-tautomer set, tautomer-mix set, overlap set, and drug-like set

Name	Unique molecules	Stable tautomers	Final size	Source
Train set	128 868	129 783	1 131 426	CSD, Tautobase, and Tautomer Database
Validation set	383	545	1539	Tautobase
Multi-tautomer set	306	614	7506	CSD
100-Tautomer set	100	142	454	Tautobase
Tautomer-mix set	140	151	698	Tautomer Database
Overlap set	762	762	11 345	DrugBank and LigandExpo
Drug-like set	2177	2177	27 357	DrugBank and LigandExpo

**Fig. 1:**
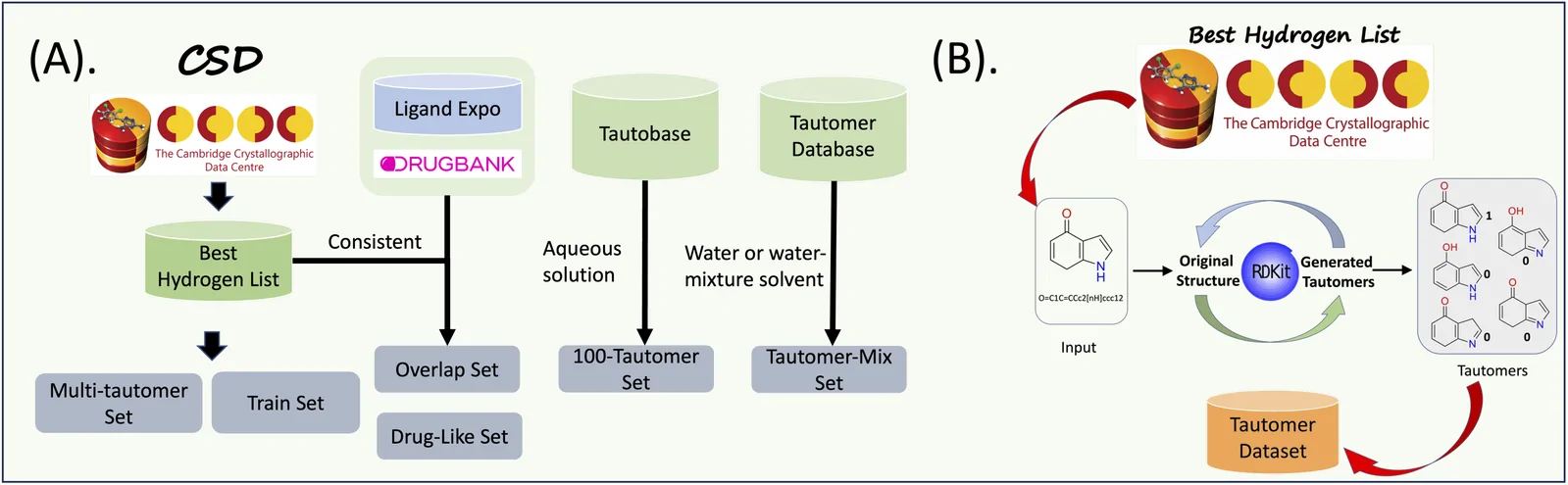
Workflow of dataset preparation. (A) Dataset assembly: the training set was created using the “Best Hydrogen List” subset of the CSD. Several distinct test sets were also assembled: an external set of molecules with multiple tautomeric states; an “overlap set” from the intersection of the CSD, DrugBank, and LigandExpo; a “drug-like set” from the intersection of the CSD with either DrugBank or LigandExpo; a “100-tautomer set” of molecules in aqueous solution; and a “tautomer-mix set” from a database of tautomers in aqueous or mixed-aqueous solvents. (B) Tautomer generation: the workflow involved enumerating all possible tautomeric states for a given molecule using the transformation rules in RDKit, followed by label assignment.

#### The tautomeric dataset from the Cambridge Structural Database

The Cambridge Structural Database (CSD) is a high-quality repository of crystal structures, many of which contain explicitly resolved hydrogen atoms for organic small molecules. Because explicit hydrogen positions identify the tautomeric form adopted in the crystal structure, these data provide direct experimental supervision for learning tautomer preferences in the solid state. Specifically, we employed the “Best Hydrogen List”,^35^ a curated subset of the CSD featuring structures with highly reliable hydrogen positions, primarily determined through neutron diffraction studies. All crystal structures from this subset were extracted using the CSD Python API, converted to SMILES, and processed with RDKit to remove invalid or charged molecules as well as molecules overlapping with any test set, resulting in 1 131 426 entries. We further identified molecules exhibiting multiple tautomeric states from this subset (Fig. 2) to form an external test set, termed the multi-tautomer set. This set challenges the model's ability to predict all stable tautomers because molecules with multiple tautomeric states were excluded from training. In addition, because this test set is derived from crystal structures, it helps evaluate model performance for predicting stable tautomers in the crystalline environment. To better simulate real-world applications, we also constructed an overlap set and a drug-like set by cross-referencing molecules from DrugBank and LigandExpo with the Best Hydrogen List and retaining those with consistent tautomeric states. This yielded 762 stable tautomeric states for the overlap set and 2177 stable tautomeric states for the drug-like set. The overlap set contains molecules with consistent tautomeric states across all three databases (CSD, DrugBank, and LigandExpo). The drug-like set contains molecules with consistent tautomeric states across any two databases (CSD and DrugBank or CSD and LigandExpo). This set is considered more reliable, as its tautomers are supported by both CSD and external databases.

**Fig. 2:**
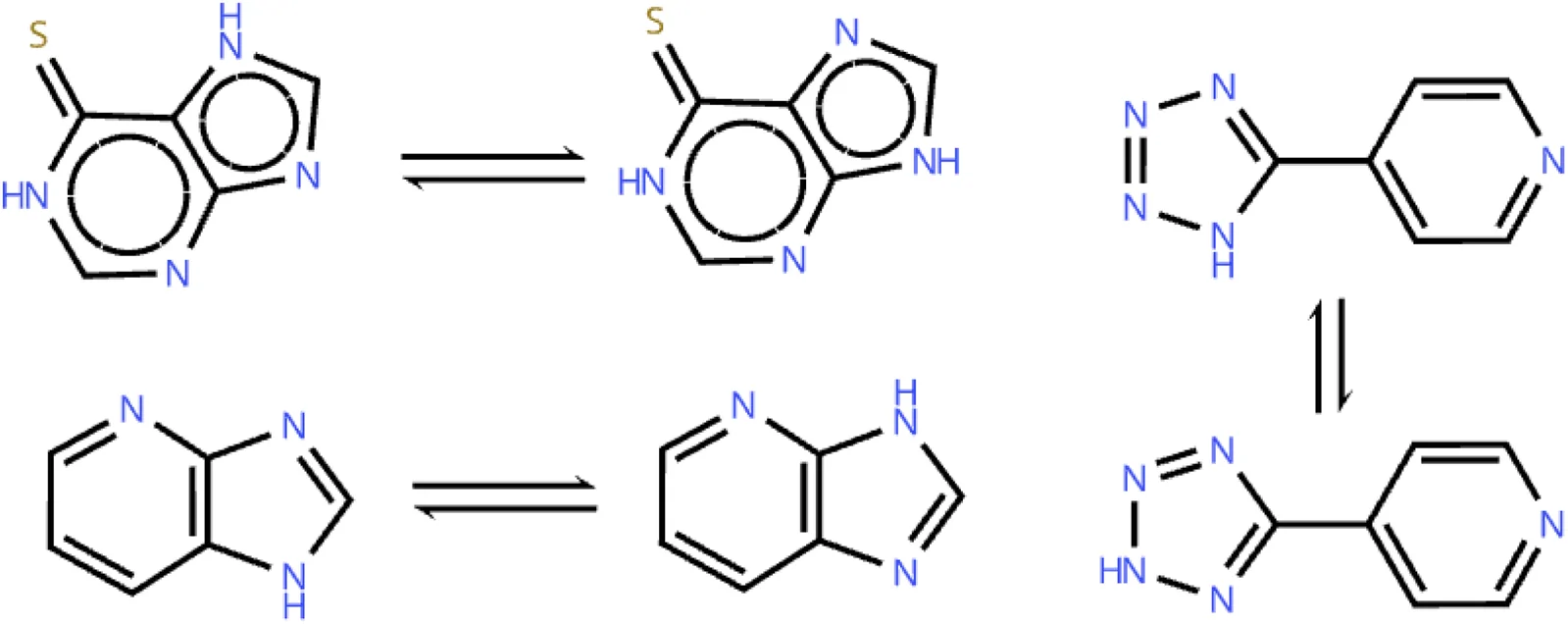
Examples of molecules from the multi-tautomer set that display multiple tautomeric states.

#### The tautomeric dataset in aqueous solution from Tautobase

In this study, our goal is to develop a model for predicting stable tautomeric states to accelerate drug discovery, focusing on water as the solvent because the most abundant tautomer in aqueous solution is most likely to bind to the target receptor. Because our model was trained mainly on crystal-structure data, we required a high-quality test set in aqueous solution to evaluate performance in identifying stable tautomeric states. Wahl *et al.*^31^ published an open-source tautomer database, Tautobase, containing experimentally measured and estimated tautomer ratios for 1680 entries in various solvents, primarily water. In a previous study, we obtained a cleaned experimental dataset for fine-tuning our model in aqueous solution, containing 483 records with log *K* values.^28^ Here, we used this dataset as the validation set for model training; it contains a total of 1539 tautomeric states after enumerating all potential tautomers using RDKit. In addition, we constructed an external test set from Tautobase, termed the 100-tautomer set. It contains 100 records with log *K* values in aqueous solution and, after RDKit enumeration, comprises 142 stable tautomers and 312 unstable tautomers. We described the data collection rules in our previous study. For these tautomer pairs, we assigned labels based on the experimental tautomer ratios. If the pairwise relative free energy satisfied |ΔΔ*G*| ≤ 2.76 kcal mol^−1^, we labeled both tautomeric states as stable. If ΔΔ*G* > 2.76 kcal mol^−1^, we labeled tautomer 1 as stable. If ΔΔ*G* < −2.76 kcal mol^−1^, we labeled tautomer 2 as stable. All other enumerated tautomeric states were labeled as unstable. The relative free energy cutoff of 2.76 kcal mol^−1^ corresponds to a 1% aqueous tautomer ratio and was used here as the criterion for assigning stable and unstable labels in aqueous-solution.^21^

#### The tautomeric dataset in water and water-containing mixed solvents from the Tautomer Database

Dhaked *et al.* collected an experimental tautomer database from the literature (the Tautomer Database), which contains 2819 tuples and 77 distinct tautomeric transformations. This dataset is valuable for evaluating our model's ability to identify stable tautomeric states in aqueous solution. For each molecule, the database provides a qualitative prevalence label: 0 (not observed), 1 (less stable), 2 (equally stable), 3 (more stable), and 4 (only observed). These labels indicate whether a tautomeric state is stable and allow the straightforward construction of a test set. Specifically, we labeled states with label 1 as unstable and states with labels 2–4 as stable. We collected all records measured in water or water-mixture solvents, resulting in 140 records and 293 structures. We then used RDKit to enumerate all tautomeric states, labeled the Tautomer Database structures as stable based on the prevalence labels, and labeled all other enumerated tautomers as unstable. This procedure yielded a total of 698 tautomeric states for these records.

### Deep learning models

To accelerate the prediction of stable tautomeric states, we trained three 2D graph-based deep learning models, MolMCL,^36^ AttentiveFP,^37^ and D-MPNN,^38–40^ as well as a baseline model, MorganFP-ANN, which uses Morgan fingerprints and a multilayer perceptron (MLP). These models leverage different levels of molecular information to learn tautomeric stability from the Cambridge Structural Database to identify stable tautomeric states for organic small molecules (as shown in Fig. 3). The training set was constructed from the CSD and experimental databases, whereas the validation set was constructed from experimental aqueous-solution data only; this enables selection of a model that performs well for drug-discovery applications in aqueous environments. For each model, we swept the probability threshold on the validation set and selected the operating threshold that maximized the *F*1 score (Table S1). The external test sets were reserved strictly for final evaluation.

**Fig. 3:**
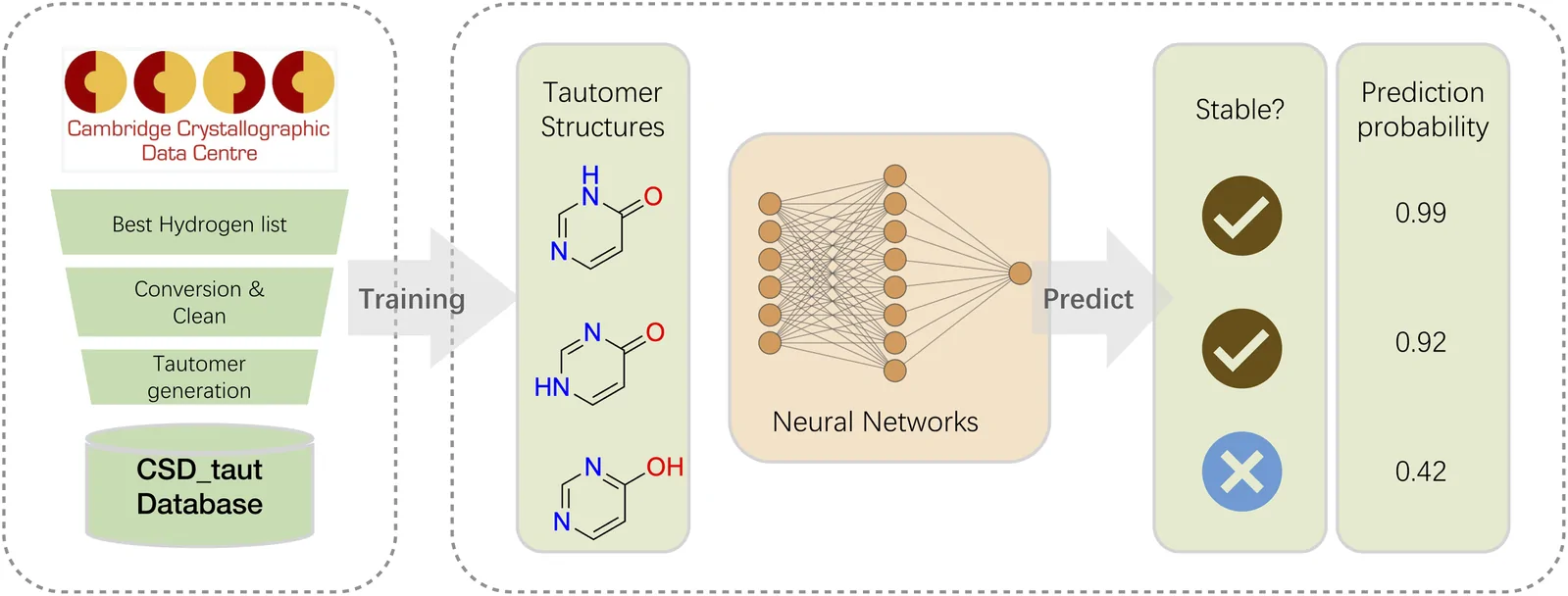
Schematic workflow of this study. First, we constructed the largest dataset from the CSD for stable-tautomer prediction; unstable tautomers were generated by RDKit. Then, we trained a graph-based classification model to learn tautomer stability.

#### MorganFP-ANN

The Morgan fingerprint^41^ is a widely used descriptor for small molecules, and its ability to represent molecules has been demonstrated in many studies (*e.g.*, property prediction, virtual screening, and similarity calculations). In this study, we combined Morgan fingerprints with an artificial neural network (ANN) as a baseline model to compare against the graph-based models and assess whether graph representations improve tautomer stability prediction. The fingerprint size was set to 2048 bits and the radius was set to 2. We used a simple multilayer perceptron (MLP) for binary classification: input features of size 2048 pass through four hidden layers with sizes 1024, 512, 256, and 128; each hidden layer uses BatchNorm1d, ReLU, and Dropout(0.2). The output layer is a single linear layer producing a logit.

#### MolMCL

MolMCL is a pretraining framework designed to generate robust and adaptable molecular representations and to be fine-tuned for downstream molecular property prediction tasks. It uses contrastive learning to learn informative molecular representations at three levels: the whole-molecule (global) level, the scaffold (substructure) level, and the functional-group (local) level. Learning these three levels of chemical knowledge involves three components: molecule contrastive learning, scaffold contrastive learning, and context prediction. In molecule contrastive learning, the model minimizes the distance between an anchor molecule and a positive molecule generated *via* subgraph masking, while maximizing the distance between the anchor molecule and negative molecules. An adaptive margin loss is used to set the separation based on fingerprint similarity. In scaffold contrastive learning, the model minimizes the distance between an anchor molecule and molecules generated by scaffold-invariant perturbations, while maximizing the distance between the anchor molecule and molecules with different scaffolds (again using an adaptive margin loss). In context prediction, the model performs masked subgraph prediction and motif prediction to determine whether a molecule contains specific functional groups. Each contrastive-learning encoder forms a channel of the overall model. During fine-tuning for a specific task, the framework learns to combine information from these channels in a context-dependent manner. The model is reported to be more robust than alternative methods by better preserving the chemical knowledge learned during pretraining, reducing overfitting and catastrophic forgetting during fine-tuning. For our task, we fine-tuned the pretrained model on our dataset for binary classification.

#### AttentiveFP

AttentiveFP is a graph neural network for molecular property prediction that extracts nonlocal intramolecular information. It builds both atom-level and molecule-level “fingerprints” using stacked attention layers, allowing each embedding to aggregate the most relevant information from neighboring atoms. At each layer, an atom aligns with its neighbors (atom and bond features) through a linear transformation and nonlinear activation to obtain a fixed-length vector, computes softmax attention weights to focus on the most relevant local context, and updates its hidden state *via* a gated recurrent unit (GRU). This design allows information to persist across hops while mitigating oversmoothing. Unlike graph models that use global mean/sum pooling for molecule-level representation, AttentiveFP constructs a virtual “supernode” and applies the same attention/GRU scheme to aggregate all atom states into a whole-molecule embedding. Architecturally, this provides three key advantages: (i) learned, anisotropic neighborhood aggregation (rather than uniform averaging) that respects molecular topology while capturing long-range, nonlocal effects; (ii) concise but expressive featurization with explicit chirality/stereochemistry and bond stereochemistry; and (iii) inherent interpretability *via* attention weights that highlight task-relevant substructures.

#### D-MPNN

The Directed Message Passing Neural Network (D-MPNN) enhances molecular representation learning by shifting from a traditional atom-centric approach to a directed bond-centric framework. In this architecture, the model iteratively updates the hidden states of directed edges. A key design choice is to exclude the reverse edge during message passing. When calculating the new state for the directed bond v → w, the network aggregates messages from all neighbors of v except w. By explicitly ignoring this immediate return signal, the model prevents redundant backtracking and encourages information propagation across the broader molecular graph. These enriched bond features are subsequently aggregated into atomic embeddings, which are then pooled to construct a holistic molecular embedding for end-to-end property prediction. The final model was trained using ChemProp v2 python package.^40^

### Workflow for the analysis of tautomeric states in PDBbind

The PDBbind dataset is widely used for developing protein–ligand scoring functions and free energy calculation methods. To systematically evaluate the utility of our model in drug discovery, we applied it to the PDBbind v2020 dataset and compared the hydrogen-bonding networks of the tautomeric states deposited in PDBbind with those of the stable tautomers predicted by our model. For ionic molecules, tautomerism is often strongly coupled to protonation/deprotonation state, so the relevant species are not simply alternative neutral tautomers, but rather a set of coupled protomer–tautomer states whose populations depend on pH and local environment. Because our current model does not explicitly enumerate or rank these coupled states, we isolated tautomerization from protonation/deprotonation changes by restricting our analysis to formally neutral, non-zwitterionic ligands. Here, we used RDKit to assess ligand neutrality, and any ligand containing an atom with a nonzero formal charge was excluded. We further curated the dataset by excluding complexes with metal ions in the immediate vicinity of the ligand, thereby avoiding the confounding effects of metal–ligand coordination, and by removing structures containing ligands with more than 35 heavy atoms. For the curated dataset, we reassigned the tautomeric states and quantified both hydrogen bonds and unsatisfied polar atoms using the Schrödinger Python API. Differences in the numbers of hydrogen bonds and unsatisfied polar atoms provide useful metrics for assessing the quality of protein–ligand complexes. When our model predicted multiple stable tautomers for a given ligand, we selected the tautomeric state with the largest number of hydrogen bonds and the fewest unsatisfied polar atoms. When these two metrics led to conflicting rankings, priority was given to the tautomeric state with the larger number of hydrogen bonds. If multiple tautomeric states showed identical numbers of hydrogen bonds and unsatisfied polar atoms, we selected the state with the highest predicted probability from our model. For all ligands with inconsistent tautomeric assignments, we further evaluated the relative energies of the original and predicted tautomeric states using DFT with an implicit solvent model. Specifically, for each molecule, an initial ensemble of 100 conformations was generated using the ETKDG algorithm in RDKit and subsequently optimized with the MMFF94 force field. The lowest-energy conformer was then selected for DFT geometry optimization at the B3LYP/6-31G(d)/SMD level. Finally, high-level single-point energies were calculated using the ωB97M-V/def2-TZVPP/SMD method. Owing to limitations of MMFF94 and B3LYP/6-31G(d), we excluded nine cases containing boron or iodine.

## Results and discussion

### Overall performance of all models across all test sets

To compare model performance and select the final model for stable tautomer prediction, we combined all external test sets into a single overall test set and evaluated overall performance on this combined test set. This set includes stable tautomeric states in aqueous solution, water-containing mixed solvents, crystal structures, and highly reliable tautomeric states for drug-like molecules from the DrugBank and LigandExpo databases, thereby providing a broad assessment of model performance in settings relevant to drug discovery. For each model, the probability threshold was first optimized on the validation set by maximizing the *F*1 score (Table S1). Models were then compared at their respective *F*1-optimized thresholds, and the final deployed model was selected by prioritizing validation-set recall. We prioritized recall because missing a truly stable tautomer—the most likely receptor-binding form—could compromise downstream docking, scoring, or free-energy calculations. By contrast, additional unstable tautomeric states can still be filtered in later scoring and ranking steps. Under this criterion, AttentiveFP was selected as the final model, achieving the highest recall on both the validation set and the overall test set, with recall values of 0.90 and 0.97, respectively. All results are summarized in Table 2, and model performance on individual test sets is provided in Tables S2–S4. Notably, the simple Morgan fingerprint-based model, MorganFP-ANN, also achieved strong performance, with recall values of 0.83 on the validation set and 0.92 on the overall test set.

**Table 2:** Performance comparison of predictive models on the validation set and all test sets. All test sets denotes the pooled test set obtained by combining all external test sets

Dataset	Model	Recall	Precision	FPR	ACC	*F*1 score	PR-AUC
Validation set	MorganFP-ANN	0.83	0.77	0.13	0.85	0.80	0.86
	MolMCL	0.86	0.78	0.13	0.87	0.82	0.87
	AttentiveFP	0.90	0.77	0.15	0.87	0.83	0.89
	D-MPNN	0.87	0.79	0.13	0.87	0.83	0.89
All test sets	MorganFP-ANN	0.92	0.84	0.02	0.98	0.88	0.95
	MolMCL	0.95	0.90	0.01	0.99	0.93	0.98
	AttentiveFP	0.97	0.88	0.01	0.99	0.92	0.98
	D-MPNN	0.96	0.90	0.01	0.99	0.92	0.98

### The performance of AttentiveFP across diverse chemical environments

Building on AttentiveFP's superior performance in our comparative analysis, we evaluated its capacity to transfer structural knowledge learned from crystal environments to aqueous solutions and diverse drug-like chemical spaces (Fig. 4). Identifying stable tautomers in aqueous solutions is critical for computational drug discovery, yet successfully generalizing models trained on solid-state crystal data (CSD) to aqueous phases remains challenging. While AttentiveFP readily captures the local atomic environments necessary to resolve solid-state stability profiles, achieving a recall of 0.87 and precision of 0.82 on the CSD-derived “multi-tautomer set”, its primary advantage is the successful transfer of this crystal-derived knowledge to aqueous domains. Encouragingly, our analysis of 625 aqueous stable tautomeric states from Tautobase revealed a 20.6% overlap (129 unique states) with the CSD neutral organic molecule set (Fig. S1), providing a rationale for this generalization. Empirically, AttentiveFP retained useful predictive performance on the aqueous-solution benchmarks, achieving recalls of 0.87 and 0.91, and precisions of 0.72 and 0.80, on the high-quality “100-tautomer set” and the broader “tautomer-mix set,” respectively. A potential limitation of the training data is that each molecule is represented by only one experimentally observed stable tautomeric state, while all other enumerated states are treated as unstable. Because some molecules may have multiple stable states, this labeling strategy may introduce noise. To assess the model under this more challenging scenario, we extracted molecules containing multiple stable tautomeric states from both the validation set and the 100-tautomer set. As shown in Table S5, the model simultaneously identified all stable tautomeric states for 68.6% of molecules in the combined subset. The corresponding success rates were 71.0% and 59.5% for the subsets derived from the validation set and the 100-tautomer set, respectively. These findings suggest that the model can identify multiple stable states in many cases, despite being trained primarily on molecules with a single annotated stable state. To assess practical utility further, we benchmarked the model against a large-scale “overlap set” and “drug-like set” curated from the CSD, DrugBank, and LigandExpo. Spanning a significantly broader chemical space than standard benchmarks, AttentiveFP delivered near-perfect classification in this regime (recall: 1.00, precision: 0.91), supporting its applicability to high-throughput screening and molecular design workflows.

**Fig. 4:**
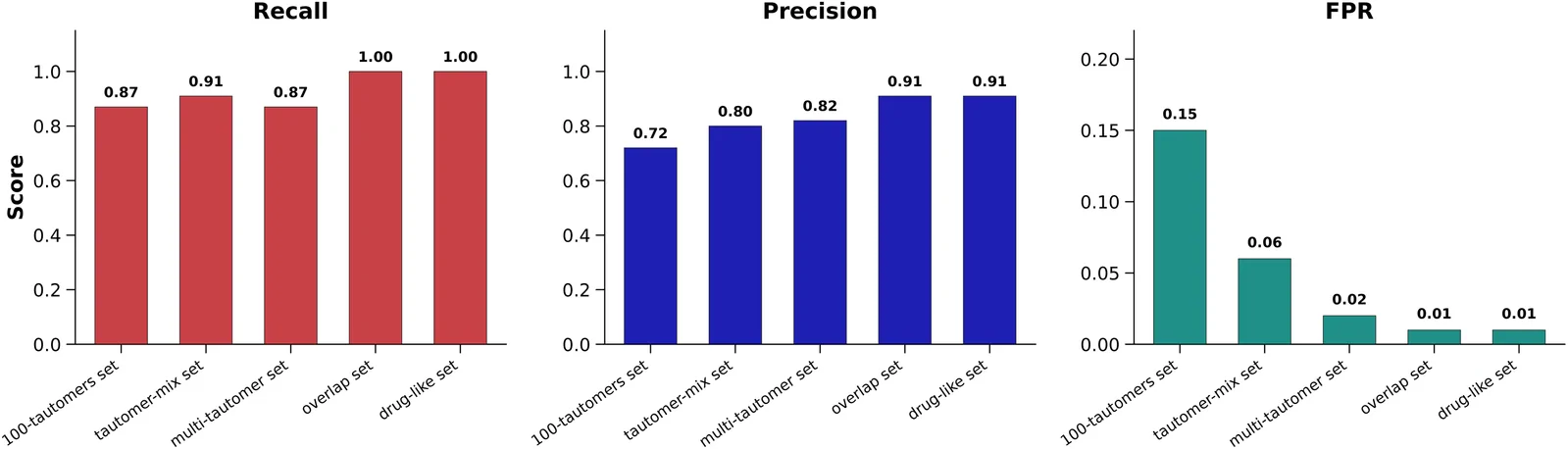
Performance of AttentiveFP on each test set, including recall, precision, and false positive rate.

However, tautomeric preferences are highly environment-dependent, and crystal structures may not always reflect the dominant tautomeric states in aqueous solution or protein-binding sites. To address this limitation, we analyzed the false-positive predictions made by AttentiveFP on both the validation set and the 100-tautomer set from aqueous solution, the distribution of these misclassification types is illustrated in Fig. 5A. The most frequent errors occurred within heteroatom H-shifts (N–O/S), endocyclic heteroatom H-shifts (ring N–N), and keto–enol/thioketo–thioenol tautomerism (Fig. 5B, rows 1–3). A representative example of this environment-dependent bias is shown in Fig. 5B (row 1), featuring the tautomeric pair of 3-acetoxypyridin-4-ol (enol form, left) and 3-acetoxypyridin-4(1*H*)-one (pyridone form, right). In Tautobase, the pyridone form (right) is recorded as the stable tautomer in aqueous solution. In contrast, AttentiveFP favors the enol form (left), which may reflect structural patterns learned from crystal-derived supervision that favor enol-like states under solid-state environments. When evaluated against aqueous test sets, this prediction was therefore classified as a false positive. These findings highlight the need to develop more precise models that account for environment-specific tautomeric preferences, particularly for these challenging tautomeric classes.

**Fig. 5:**
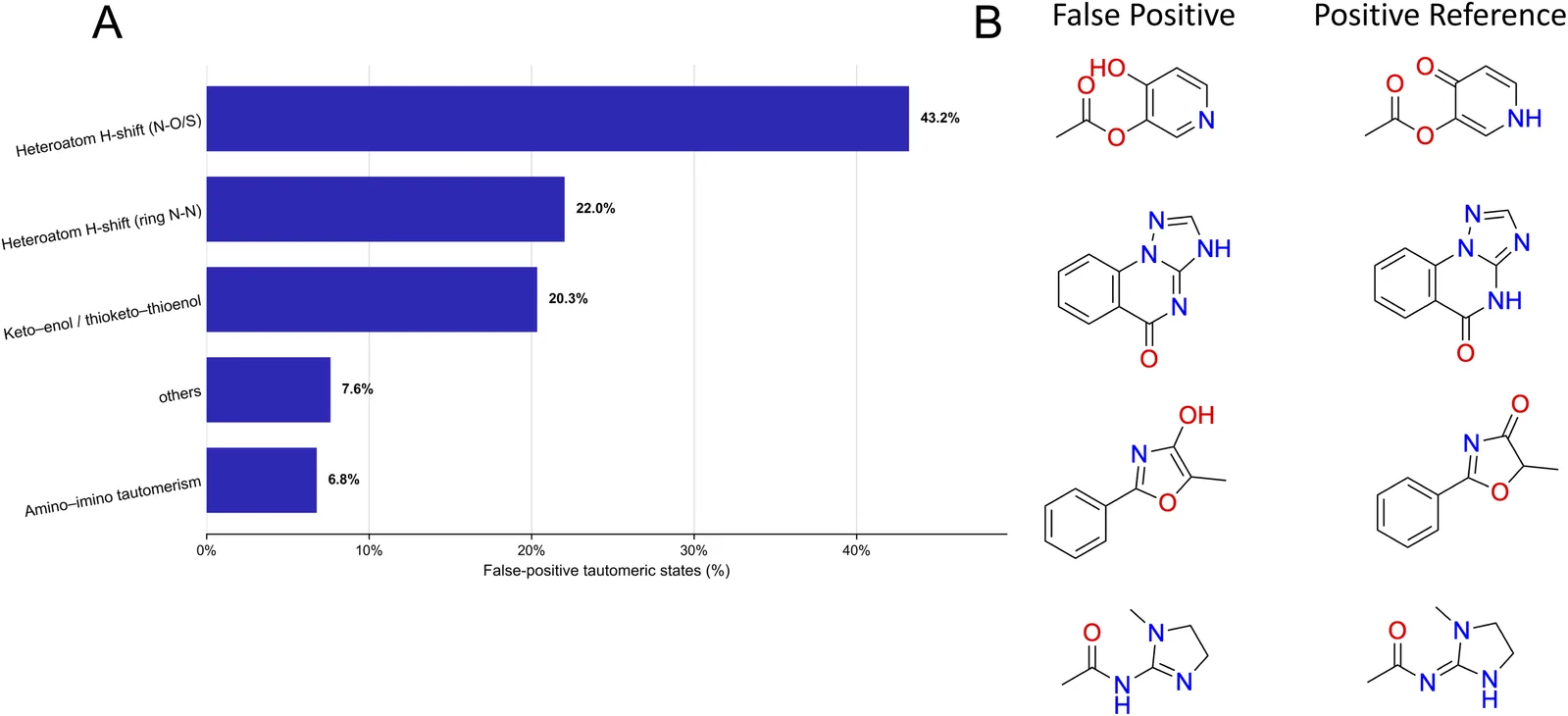
Evaluation of false-positive predictions. (A) Distribution of false-positive tautomeric states predicted by AttentiveFP across the validation and 100-tautomer datasets. (B) Representative examples of false-positive predictions across distinct tautomeric categories: heteroatom H-shift (N–O/S), ring N–N H-shift, keto–enol/thioketo–thioenol, and amino–imino tautomerism.

### Application to identify stable tautomeric states

After applying our model to the PDBbind dataset, we found that 279 of the 5075 ligands with multiple tautomeric states, out of a total of 6202 ligands without charged function groups, showed inconsistencies between the original and predicted tautomeric states. The original structures and predicted tautomeric states for these ligands are provided in the SI. We first focused on a subset of 126 ligands, here termed the structure-resolved set, in which the reassigned tautomer simultaneously increased the number of protein–ligand hydrogen bonds and reduced the number of unsatisfied polar atoms relative to the deposited state. These cases provide the strongest structural support that the original tautomeric assignment is likely incorrect. As summarized in Fig. 6A, reassignment in this high-confidence set (green color) consistently improved the chemical reasonableness of the bound ligand state. To further assess the consequences for structure-based modeling, we calculated Glide SP docking scores for these 126 structures and compared the resulting scores with experimental binding affinities. The reassigned tautomeric states showed a lower mean absolute error between experimental binding affinities and Glide SP scores than the original states (MAE = 1.65 kcal mol^−1^*vs.* 2.30 kcal mol^−1^) and yielded more favorable predicted binding free energies (Fig. 6B and C). Together, these results indicate that tautomer reassignment can materially improve both local interaction patterns and docking-based evaluation in a substantial subset of protein–ligand complexes. In addition, 94% of the predicted tautomeric states in this set are energetically favorable compared to the original states based on DFT calculations. Furthermore, with 98% of the calculated relative energies remaining within a 2.76 kcal mol^−1^ margin (Fig. 7A), these energetic evaluations provide additional support for the predicted tautomer assignments.

**Fig. 6:**
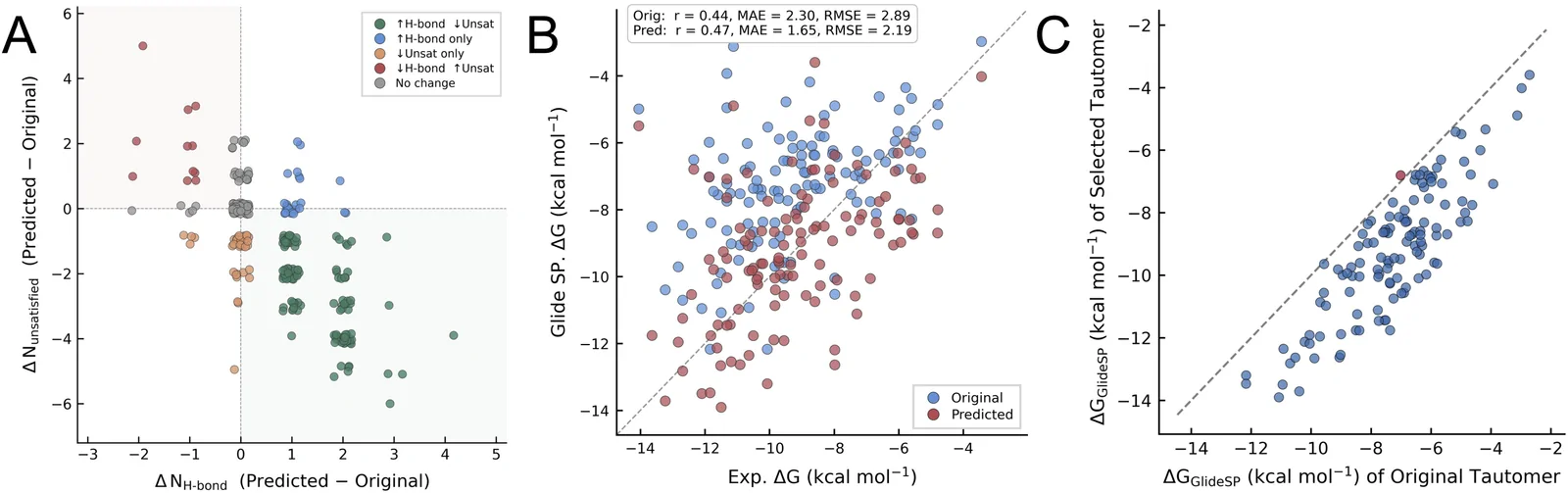
(A) Changes in hydrogen-bond count and the number of unsatisfied polar atoms between the original PDBbind v2020 tautomer and the predicted stable tautomer (*N*_predicted_ − *N*_original_). For ligands with multiple stable tautomers, the tautomer with the maximum number of hydrogen bonds and the minimum number of unsatisfied polar atoms is reported. (B) Correlation between Glide SP scores and experimental binding affinities for the original and reassigned tautomeric states. (C) Differences in Glide SP scores between the original and reassigned tautomeric states.

**Fig. 7:**
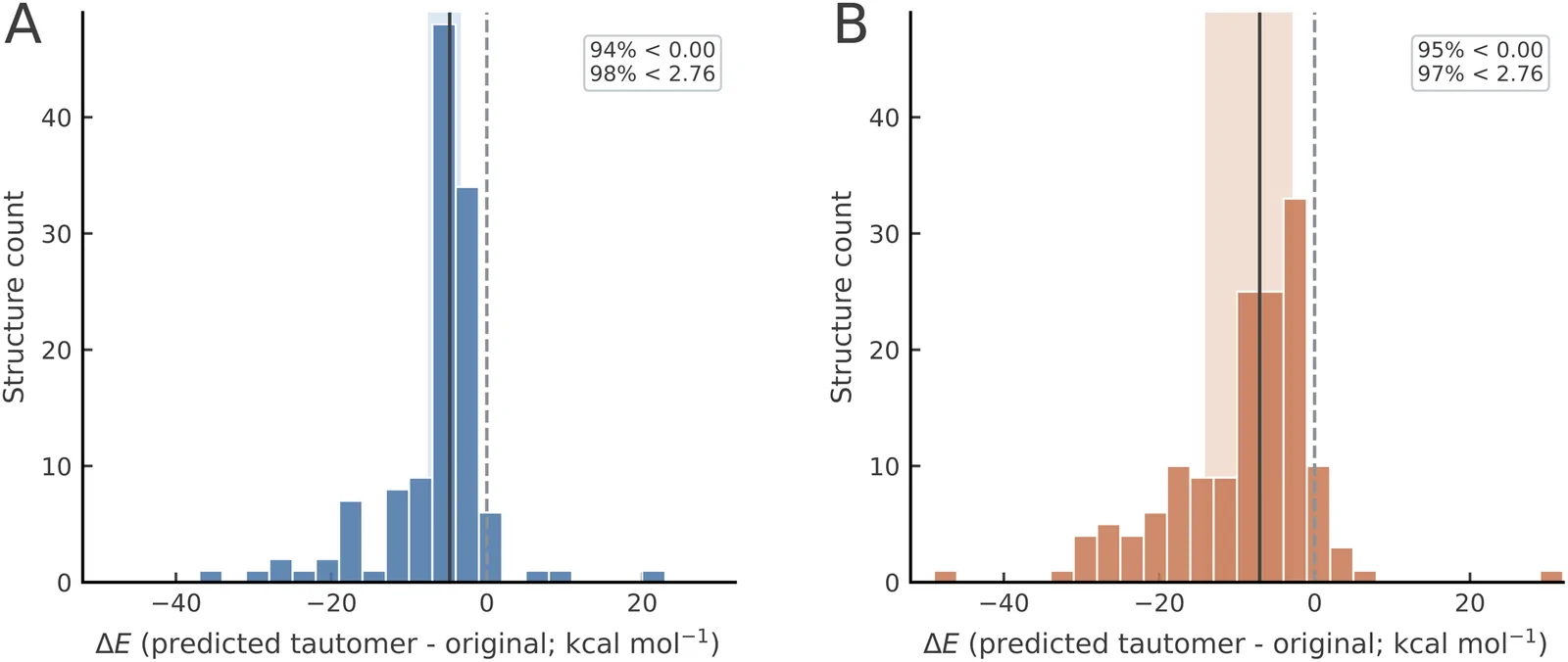
Distribution of tautomer relative energies (ωB97M-V/def2-TZVPP/SMD) of the predicted tautomer *versus* the original structure in two PDBbind-derived structure sets. Energy differences are reported as predicted tautomer minus original structure. (A) The structure-resolved set; (B) the structure-unresolved set.

The remaining 153 ligands, referred to here as the structure-unresolved set, also showed inconsistencies between the deposited and predicted tautomeric states, but reassignment did not lead to both improved hydrogen bonding and fewer unsatisfied polar atoms. We therefore further evaluated these 153 complexes using Glide SP docking. As shown in Fig. S2, the Glide SP docking scores did not differ significantly between the deposited and predicted tautomeric states. These cases may represent more complex situations, including receptor-induced stabilization of higher-energy bound tautomers, limitations of the scoring function, or tautomeric changes occurring in solvent-exposed regions where their effects on binding are less pronounced. Because the preferred tautomeric states of these ligands cannot be confidently resolved from the present analysis alone, further investigation using additional structural evidence is required. For the energy calculations, the predicted tautomeric state was energetically more favorable than the deposited state in 95% of cases, and 97% of the relative energy differences were within 2.76 kcal mol^−1^ (Fig. 7B). These results suggest that, although the predicted tautomers are generally more stable, higher-energy tautomers may still be stabilized in specific binding-pocket environments. Therefore, protein–ligand scoring functions should account not only for binding interactions but also for the energetic penalty associated with adopting a higher-energy tautomeric state in the binding pocket.

We selected two representative cases, ligand 529 in 2BPM and WF8 in 4BTX, to illustrate how tautomer reassignment can improve the interpretation of protein–ligand hydrogen-bonding networks (Fig. 8). Ligand 529 in 2BPM is a 3-aminopyrazole CDK2/cyclin A inhibitor developed as a potential anticancer agent.^42^ Its heterocyclic core can adopt two prototropic tautomeric states, the 1H and 2H forms. This distinction is important because proton transfer changes the ligand's hydrogen-bond donor and acceptor pattern, thereby affecting its interactions within the kinase binding site. Structural analysis showed that the 2H form fails to maintain key hydrogen bonds with LEU83 and GLU81, whereas these interactions are preserved in the 1H form (Fig. 8A). Consistent with this binding-mode analysis, our model assigned a high probability to the 1H form (0.99) and a lower probability to the 2H form (0.40). We also noted an inconsistency between the ligand annotation in the RCSB Protein Data Bank and the structure reported in the original literature. We further analyzed WF8 in 4BTX, a pyridazinone inhibitor bound to human vascular adhesion protein-1 (VAP-1).^43^ Pyridazinone inhibitors such as WF8 were designed to achieve potent, selective, and reversible inhibition of VAP-1 over monoamine and diamine oxidases. Exhaustive enumeration of WF8 generated 12 possible tautomeric states, among which our model identified two likely stable forms. Crystallographic evidence indicates that WF8 adopts the 2H form in the binding pocket. Comparison of the binding modes showed that the alternative 1H form fails to form an important interaction with ASP180 (Fig. 8B). Consistent with this structural evidence, our model assigned a substantially higher probability to the 2H form (0.96) than to the 1H form (0.53). Together, these examples show that accurate prediction of stable tautomeric states can improve the assignment and interpretation of hydrogen-bonding networks in protein–ligand complex structures.

**Fig. 8:**
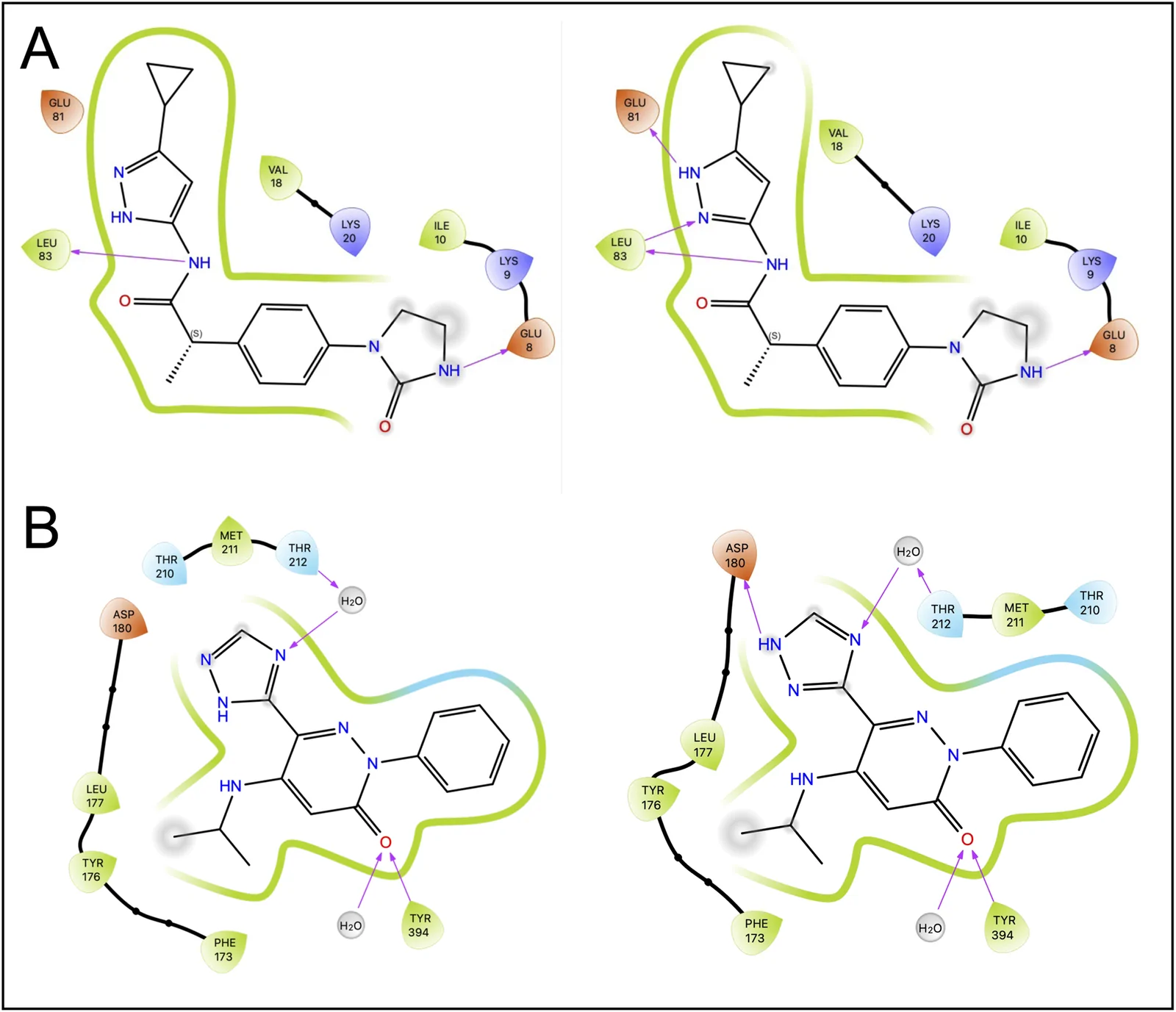
Comparison of hydrogen bonding networks between the original and reassigned tautomeric states. (A) Ligand 529, (2*S*)-*N*-(5-cyclopropyl-1*H*-pyrazol-3-yl)-2-[4-(2-oxo-1-pyrrolidinyl)phenyl]propanamide in complex of 2BPM. (B) Ligand WF8, 5-isopropylamino-2-phenyl-6-(1*H*-1,2,4-triazol-5-yl)-3(2*H*)-pyridazinone in complex of 4BTX. Hydrogen bonds are shown for the original PDBbind v2020 states *versus* the states reassigned by Tautomer-Predictor.

### Runtime on large chemical libraries

In this study, we developed an easy-to-use tool for predicting stable tautomeric states based on the trained models, termed “Tautomer-Predictor”. To evaluate the scalability of our tool for processing large chemical libraries, we applied Tautomer-Predictor to predict stable tautomeric states across several widely used databases, including the Enamine Screening Collection, ChEMBL, ChemDiv, and Asinex. Among the graph-based models, AttentiveFP demonstrated the best performance; consequently, we selected AttentiveFP as the predictive model for “Tautomer-Predictor”. All computations were performed on a system equipped with an AMD 128-core CPU and a single NVIDIA A100 GPU. Table 3 presents a comparison of the total tautomer counts derived from RDKit enumeration *versus* the stable tautomers predicted by our model, along with the corresponding running times. The results demonstrate high computational efficiency; notably, processing the Enamine collection (over 4.6 million compounds) required only 3.19 hours. Collectively, these results confirm our model's ability to process extensive chemical libraries efficiently, suggesting strong potential for applications in computational drug discovery.

**Table 3:** Computational efficiency and prediction statistics of Tautomer-Predictor on large-scale chemical libraries

Database	Size	Tautomeric molecules	All tautomers	Predicted stable tautomers	Running time
Enamine	4 671 131	3 623 149	24 102 643	3 917 535	3.19 h
ChEMBL	2 854 801	2 204 324	83 362 598	2 732 174	31.08 h
ChemDiv	1 614 271	1 293 581	8 238 810	1 392 129	0.97 h
Asinex	575 299	473 128	3 619 606	515 205	0.67 h

## Conclusion

In this study, we demonstrate that crystallographic proton positions provide a powerful large-scale source of supervision for learning tautomer stability. By mining proton-resolved structures from the Cambridge Structural Database, we constructed a dataset of more than 1.1 million tautomeric states and trained a 2D graph neural network that predicts stable tautomeric states directly from molecular topology, without conformer generation or quantum-mechanical calculations. The model showed strong performance across crystal-structure, aqueous-solution, and drug-like benchmarks, indicating that transferable and chemically meaningful rules of tautomer stability can be learned from crystallographic proton placements.

We further demonstrated the practical value of this approach for structure-based modeling. Applied to the 5075 PDBbind ligands with multiple tautomeric states, the model identified 126 cases in which the assigned tautomeric state is likely incorrect. In each of these cases, the reassigned stable tautomer exhibited improved hydrogen-bonding patterns together with fewer unsatisfied polar atoms, supporting the utility of the method for improving chemically reasonable ligand-state assignment in protein–ligand complexes. More broadly, these results highlight the importance of tautomer treatment in studying protein–ligand interactions. To enable large-scale application, we developed the open-source workflow Tautomer-Predictor, which processes the 4.6-million-compound Enamine collection in about 3.2 h on a single GPU-enabled node. Despite its robust performance, the current workflow remains most applicable to neutral organic molecules and does not explicitly account for ionic systems, metal coordination, receptor-induced tautomer stabilization, or incompleteness in RDKit tautomer enumeration. In particular, developing unified frameworks that can simultaneously resolve coupled protonation and tautomerization states remains a crucial avenue for future research.

In summary, this work establishes crystallographic proton placement as a powerful source of transferable supervision for learning tautomer stability. By mining the Cambridge Structural Database at scale, we trained a 2D graph neural network that predicts stable tautomeric states directly from molecular topology, combining strong predictive performance with the speed required for modern molecular discovery. The model shows encouraging performance across solid-state and aqueous-solution benchmarks, as well as diverse drug-like molecules, and its application to PDBbind shows that improved tautomer assignment can directly enhance the chemical reasonableness of protein–ligand binding models. More broadly, these results demonstrate how large-scale experimental structural data can be repurposed to learn chemically meaningful rules of molecular state preference, providing a practical framework for large-scale tautomer assignment in drug discovery.

## Author contributions

Xiaolin Pan: conceptualization, data curation, software and writing – original draft; Chao Han: data curation, analysis, and writing – review; Fengyang Han: analysis and writing – review; Yingkai Zhang: conceptualization, supervision, writing – review & editing.

## Conflicts of interest

There are no conflicts to declare.

## Supplementary Material

SC-017-D6SC03714C-s001

SC-017-D6SC03714C-s002

SC-017-D6SC03714C-s003

## Data Availability

The dataset and source code for data preparation, model training, and model usage are available at https://github.com/xiaolinpan/Tautomer-Predictor. Due to CSD license restrictions, we only publish the entry ID for each molecule from the “Best Hydrogen List” subset. The structural comparisons between the original and predicted tautomeric states in the PDBbind v2020 dataset are provided in the supplementary information (SI) as pdbbind_tautomers_structures.xlsx. Supplementary information: Text S1: detailed description of the evaluation metrics used for model performance assessment; Fig. S1: intersection of the CSD and stable tautomeric states in aqueous solution from tautobase; Fig. S2: comparison of Glide SP scores and their correlation with experimental binding affinities for original *versus* predicted tautomeric states; Table S1: best probability cutoff selected on the validation set for each predictive model; Table S2: performance metrics for fingerprint-based and graph-based models evaluated on the multi-tautomer dataset; Table S3: performance comparison of predictive models on the aqueous 100-tautomer set and the tautomer-mix set; Table S4: performance comparison of predictive models on the overlap set and drug-like set; Table S5: performance on molecules with multiple experimentally annotated stable tautomeric states from the validation set and the 100-tautomer set; false_positive_molecule.xlsx: false positive molecules and true positives from validation set and 100-tautomer set; pdbbind_tautomers_structures.xlsx: structural comparison between the original and predicted tautomeric states in the PDBbind v2020 dataset. See DOI: https://doi.org/10.1039/d6sc03714c.
