# Supplementary material for: Deep learning of tautomer stability from crystallographic proton positions
Source: Chem Sci. 2026 Sep 23. Online ahead of print. doi: 10.1039/d6sc03714c (PMC13598646; doi:10.1039/d6sc03714c)
Supplement: Supplementary file 1 [file SC-OLF-D6SC03714C-s003.pdf]

Supplementary Information (SI) for Chemical Science.
This journal is © The Royal Society of Chemistry 2026

Supporting Information: Deep learning of tautomer stability from crystallographic proton positions

Xiaolin Pan,[†] Chao Han,[†] Fengyang Han,[†] and Yingkai Zhang[*,†,‡]

†*Department of Chemistry, New York University, New York 10003, United States*

‡*Simons Center for Computational Physical Chemistry at New York University, New York, New York 10003, United States*

E-mail: Yingkai.zhang@nyu.edu

Table of Contents

Text S1. Evaluation metrics **S-3**

Table S1. Best probability cutoff selected on validation set. **S-3**

Table S2. Performance metrics for fingerprint-based and graph-based models evaluated on the multi-tautomer dataset. **S-4**

Table S3. Performance comparison of predictive models on the aqueous "100-tautomer set" and the "tautomer-mix set". **S-4**

Table S4. Performance comparison of predictive models on overlap set and drug-like set. **S-4**

Table S5. Performance on molecules with multiple experimentally annotated stable tautomeric states from the validation set and the 100-tautomer set. **S-4**

Figure S1. Intersection of the CSD and Tautobase datasets. **S-5**

Figure S2. Comparison of Glide SP scores and their correlation with experimental binding affinities for original versus predicted tautomeric states. **S-6**

Text S1. Evaluation metrics

In this study, we evaluated the classification performance of our model using several commonly used metrics, including accuracy (ACC), recall, F1 Score, and the area under the precision–recall curve (PR-AUC). Given that the dataset is highly imbalanced, PR-AUC was additionally employed to provide a more reliable assessment of model performance, as it focuses on the trade-off between precision and recall rather than overall accuracy. The metrics are defined as follows:

$$\text{Recall} = \frac{TP}{TP + FN}, \quad \text{Precision} = \frac{TP}{TP + FP} \tag{1}$$

$$\text{F1-Score} = 2 \times \frac{\text{Precision} \times \text{Recall}}{\text{Precision} + \text{Recall}}, \tag{2}$$

$$\text{PR-AUC} = \int_0^1 \text{Precision}(r)\, dr \tag{3}$$

$$\text{FPR}(t) = \frac{FP}{FP + TN} \tag{4}$$

where TP, TN, FP, and FN denote the numbers of true positives, true negatives, false positives, and false negatives, respectively. PR-AUC measures the area under the precision-recall curve, where precision is evaluated as a function of recall across different classification thresholds.

Table S1: Best probability cutoff selected on validation set.

**Model**	**Probability cutoff**
MorganFP-ANN	0.38
MolMCL	0.40
AttentiveFP	0.43
D-MPNN	0.08

Table S2: Performance metrics (Recall, Precision, False Positive Rate, F1 Score, and PR-AUC) for fingerprint-based and graph-based models evaluated on the multi-tautomer dataset.

**Model**	**Recall**	**Precision**	**FPR**	**ACC**	**F1 Score**	**PR-AUC**
MorganFP-ANN	0.67	0.85	0.01	0.96	0.75	0.83
MolMCL	0.78	0.86	0.01	0.97	0.82	0.92
AttentiveFP	0.87	0.82	0.02	0.97	0.85	0.91
D-MPNN	0.81	0.87	0.01	0.97	0.84	0.92

Table S3: Performance comparison of predictive models on the aqueous ”100-tautomer set” and the ”tautomer-mix set”.

**Dataset**	**Model**	**Recall**	**Precision**	**FPR**	**ACC**	**F1 Score**	**PR-AUC**
100-tautomer set	MorganFP-ANN	0.82	0.74	0.13	0.85	0.78	0.81
	MolMCL	0.86	0.74	0.13	0.86	0.80	0.84
	AttentiveFP	0.87	0.72	0.15	0.85	0.79	0.87
	D-MPNN	0.84	0.75	0.13	0.86	0.79	0.87
tautomer-mix set	MorganFP-ANN	0.83	0.84	0.04	0.93	0.84	0.89
	MolMCL	0.86	0.85	0.04	0.94	0.86	0.85
	AttentiveFP	0.91	0.80	0.06	0.93	0.85	0.90
	D-MPNN	0.86	0.87	0.03	0.94	0.87	0.91

Table S4: Performance comparison of predictive models on overlap set and drug-like set.

**Dataset**	**Model**	**Recall**	**Precision**	**FPR**	**ACC**	**F1 Score**	**PR-AUC**
Overlap set	MorganFP-ANN	0.98	0.85	0.01	0.99	0.91	0.99
	MolMCL	0.99	0.92	0.01	0.99	0.96	1.00
	AttentiveFP	1.00	0.91	0.01	0.99	0.95	1.00
	D-MPNN	0.99	0.91	0.01	0.99	0.95	0.99
drug-like set	MorganFP-ANN	0.98	0.84	0.02	0.98	0.91	0.98
	MolMCL	1.00	0.92	0.01	0.99	0.96	1.00
	AttentiveFP	1.00	0.91	0.01	0.99	0.95	1.00
	D-MPNN	1.00	0.91	0.01	0.99	0.95	1.00

Table S5: Performance on molecules with multiple experimentally annotated stable tautomeric states from the validation set and the 100-tautomer set.

**Original**	**Recall**	**Precision**	**FPR**	**ACC**	**F1 Score**	**Success Rate**
Validation set	0.85	0.84	0.12	0.87	0.84	71.0% (115/162)
100-tautomer set	0.79	0.83	0.10	0.86	0.81	59.5% (25/42)
Total	0.83	0.84	0.12	0.86	0.83	68.6% (140/204)

Note: “Success rate” denotes the fraction of molecules for which all experimentally annotated stable tautomeric states were correctly identified simultaneously.

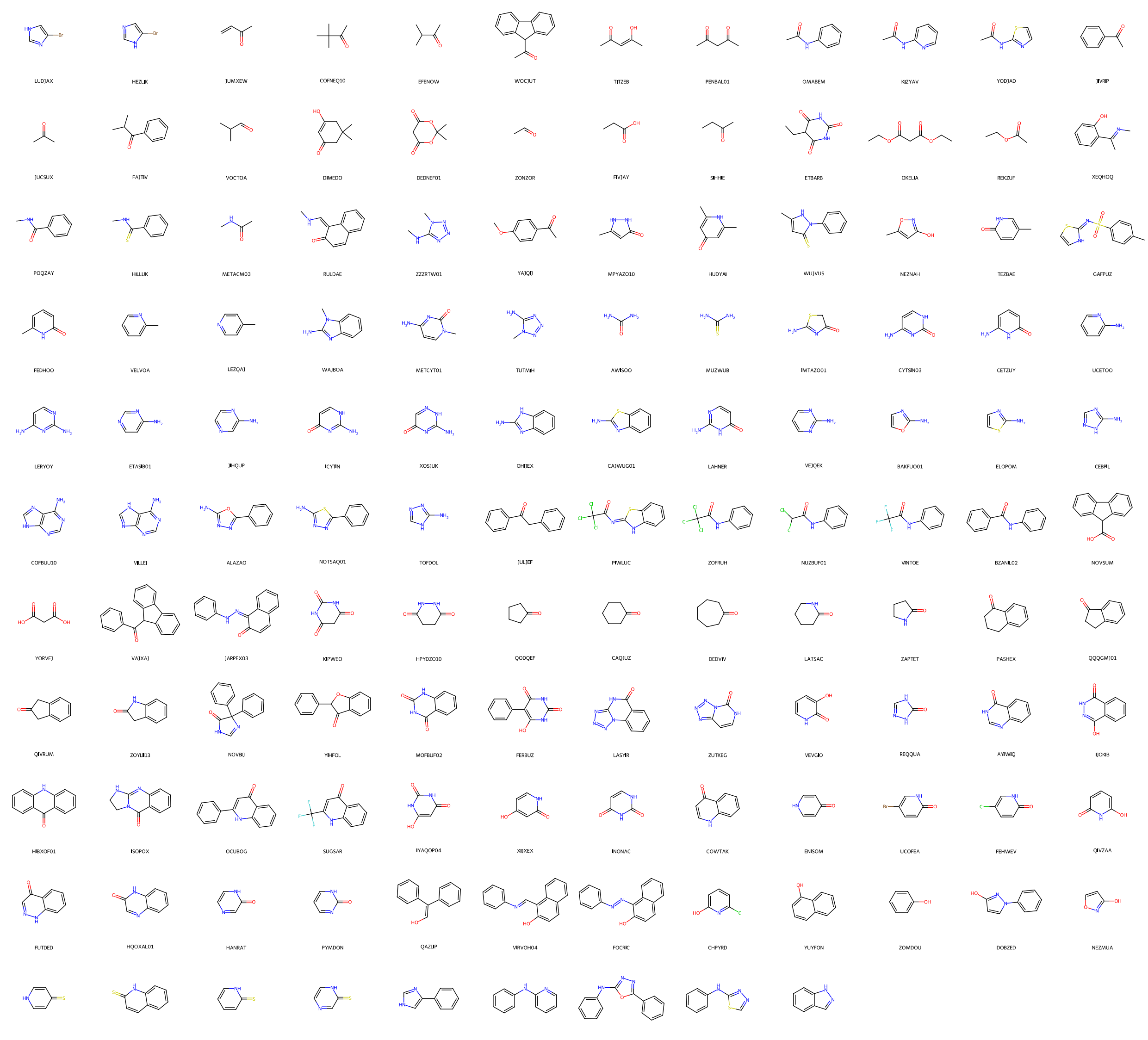

Figure S1: Intersection of the CSD and Tautobase datasets. The set comprises 129 unique molecules that exist in both crystal structures (with assigned CSD Refcodes) and aqueous solution.

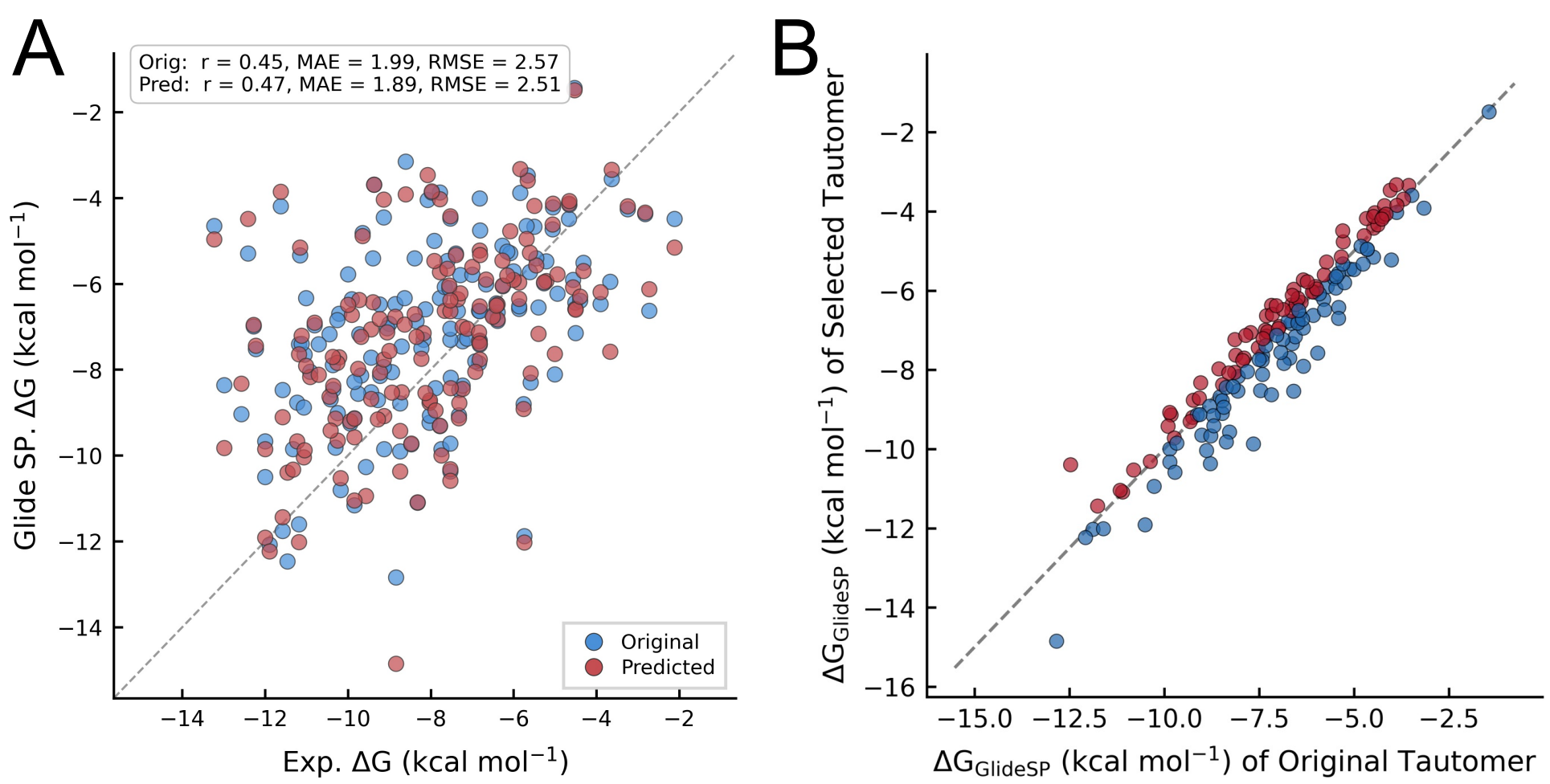

Figure S2: (A) Correlation of Glide SP scores with experimental binding affinities for original versus predicted tautomeric states. (B) Difference in Glide SP scores between the original and predicted tautomeric states. Note: Both plots exclude complexes that simultaneously exhibit a decrease in unsatisfied polar atoms and an increase in hydrogen bonds.
